# Phylogenetic study of six species of *Anopheles* mosquitoes in Peninsular Malaysia based on inter-transcribed spacer region 2 (ITS2) of ribosomal DNA

**DOI:** 10.1186/1756-3305-7-309

**Published:** 2014-07-03

**Authors:** Jia-Siang Sum, Wenn-Chyau Lee, Amirah Amir, Kamil A Braima, John Jeffery, Noraishah M Abdul-Aziz, Mun-Yik Fong, Yee-Ling Lau

**Affiliations:** 1 Tropical Infectious Disease Research and Education Center (TIDREC), Department of Parasitology, Faculty of Medicine, University of Malaya, Kuala Lumpur 50603, Malaysia

**Keywords:** *Anopheles*, Peninsular Malaysia, ITS2 rDNA, Phylogenetic study, Vicariance, Geographical segregation

## Abstract

**Background:**

Molecular techniques are invaluable for investigation on the biodiversity of *Anopheles* mosquitoes. This study aimed at investigating the spatial-genetic variations among *Anopheles* mosquitoes from different areas of Peninsular Malaysia, as well as deciphering evolutionary relationships of the local *Anopheles* mosquitoes with the mosquitoes from neighbouring countries using the anopheline ITS2 rDNA gene.

**Methods:**

Mosquitoes were collected, identified, dissected to check infection status, and DNA extraction was performed for PCR with primers targeting the ITS2 rDNA region. Sequencing was done and phylogenetic tree was constructed to study the evolutionary relationship among *Anopheles* mosquitoes within Peninsular Malaysia, as well as across the Asian region.

**Results:**

A total of 133 *Anopheles* mosquitoes consisting of six different species were collected from eight different locations across Peninsular Malaysia. Of these, 65 ITS2 rDNA sequences were obtained. The ITS2 rDNA amplicons of the studied species were of different sizes. One collected species, *Anopheles sinensis*, shows two distinct pools of population in Peninsular Malaysia, suggesting evolvement of geographic race or allopatric speciation.

**Conclusion:**

*Anopheles* mosquitoes from Peninsular Malaysia show close evolutionary relationship with the Asian anophelines. Nevertheless, genetic differences due to geographical segregation can be seen. Meanwhile, some *Anopheles* mosquitoes in Peninsular Malaysia show vicariance, exemplified by the emergence of distinct cluster of *An. sinensis* population.

## Background

*Anopheles* mosquitoes are one of the most studied members of the Culicidae family. The discovery of *Anopheles* as the exclusive vector for malaria transmission in humans has garnered much attention to study this particular genus. Due to insecticide usage in malaria control programs and agricultural practices, *Anopheles* mosquitoes are subjected to high mutation rate and selective pressure [1-3]. Besides, geographical barriers such as mountains and seas cause vicariance [4], thus preventing genetic interchange among *Anopheles* of the same species from different locations or countries. Occasionally, these phenomena drive speciation, where the new population shows biological characteristics that are different from the parent species [5,6]. Such biological differences include degree of resistance against insecticides, susceptibility to malaria parasites and capability in malaria transmission. In view of this, the population and evolutionary dynamics of *Anopheles* mosquitoes deserve research attention, especially in countries that are at the edge of complete malaria eradication, exemplified by Malaysia.

Molecular technique provides a powerful tool for effective investigation on the population dynamics of mosquitoes. It enables more detailed understanding on the relationships between the vectorial capacity, genetic makeup and geographical origin for a particular species of *Anopheles*, more detailed and precise taxonomy, as well as evolutionary studies [6-10]. Various gene markers have been selected for such study purposes. Gene sequences such as Internal Transcribed Spacer 1 and 2 (ITS1 & 2) of ribosomal DNA (rDNA), mitochondrial cytochrome c oxidase subunit I and II (COI & COII), and D3 (28S rDNA) are helpful in species identification and phylogenetic analyses [10-25]. Among these gene sequences, the ITS2 of rDNA (ITS2 rDNA) has been found to be valuable for taxonomic classification [24,26-29]. ITS2 rDNA is a non-coding DNA sequence. Therefore, it is subjected to a high degree of mutations, which makes it a good candidate to study phylogenetics of closely related *Anopheles* species, as well as biodiversity and geographic races of a particular species of mosquitoes [30]. To date, the ITS2 rDNA sequences have been successfully used to distinguish members of several *Anopheles* species complexes, such as *An. hyrcanus* group [24], *An. dirus* complex [12] and *An. maculatus* group [31]. Regrettably, knowledge regarding Malaysian *Anopheles* population characterization based on ITS2 rDNA is not well established [31-38]. Hence, there is a need to fill this knowledge gap for better understanding on biodiversity of *Anopheles* in this region based on ITS2 rDNA.

This study aimed at characterizing the ITS2 rDNA sequences of several *Anopheles* mosquitoes in Peninsular Malaysia, as well as investigating the spatial-genetic variations among *Anopheles* mosquitoes from different areas of Peninsular Malaysia. Besides, this study also aimed at deciphering the evolutionary relationship of the local *Anopheles* mosquitoes with the mosquitoes from other Asian countries.

## Methods

### Mosquito collection and identification

This study was approved by Institutional Animal Care and Use Committee (IACUC) of University of Malaya [PAR/19/02/2013/AA (R)]. Mosquitoes were collected from twenty locations across eight states of Peninsular Malaysia using the bare-leg catch (BLC) method and human-bait net trapping method as described previously [39,40]. Collection sites were selected based on previous studies [41,42], as well as information regarding malaria case incidence provided by the District Health Offices of respective states. Collection was conducted from hour 1800 to 2330. The captured mosquitoes were kept in a glass tube containing moist tissue for further processing in the laboratory. *Anopheles* mosquitoes were sorted from the collected mosquitoes, subsequently differentiated into species based on taxonomy morphological keys, with the aid of a stereomicroscope as described previously [43,44]. Dissection, coupled with polymerase chain reaction (PCR) was performed on the captured *Anopheles* mosquitoes to investigate the infection status by the malaria parasites as described in a previous study [45].

### Mosquito DNA extraction and amplification

DNA extraction was conducted with DNeasy® Blood & Tissue Kit (QIAGEN, USA) according to manufacturer’s instructions. The final DNA product was dissolved in 60 μL elution buffer and stored at −20°C until PCR analysis.

Sequences of ITS2 rDNA from extracted DNA of each *Anopheles* mosquito were amplified using the primers and protocols developed previously [12]. Briefly, reactions were performed using MyCycler™ Thermal Cycler (Bio-Rad, USA). Each reaction mixture of 25 μL contained 4 μL of mosquito DNA template, primers ITS2A (5’ TGT GAA CTG CAG GAC A 3’) and ITS2B (5’ TAT GCT TAA ATT CAG GGG GT 3’), 0.2 μM respectively, 0.2 mM dNTP, 4 mM MgCl_2_, 10 μL of GoTaq® Flexi Buffer and 1.25 U of GoTaq® DNA polymerase (Promega, USA). The PCR conditions were as follows: (1) denaturation at 94°C for 5 minutes, (2) 35 cycles of amplification at 94°C for 1 minute, annealing step at 51°C for 1 minute with elongation step at 72°C for 2 minutes, followed by (3) final elongation step of 10 minutes at 72°C and a hold temperature of 4°C.

### DNA sequencing and analysis

The PCR amplicons were ligated to pGEM®-T vector (Promega, USA) and transformed into One Shot® TOP10 *Escherichia coli* competent cells (Invitrogen™, USA). Recombinant plasmid was extracted and purified using QIAprep® Spin Miniprep Kit (Qiagen, USA). ITS2 rDNA was sequenced using the M13 forward (−20) and reverse (−24) universal sequencing primers. Sequences were edited using UGENE software and aligned in ClustalW program using the default parameters. By using Basic Local Alignment Search Tool (BLAST) [46], sequence identity comparison and confirmation were carried out using gene sequence read archive (SRA) of GenBank. Subsequently, multiple sequence alignment of ITS2 rDNA was conducted and Neighbor-Joining (bootstrap = 1000) [47] and Maximum Parsimony analysis [48] were employed to study the evolutionary relationship among *Anopheles* mosquitoes within Malaysia, as well as across the Asian region.

## Results

**Figure 1:**
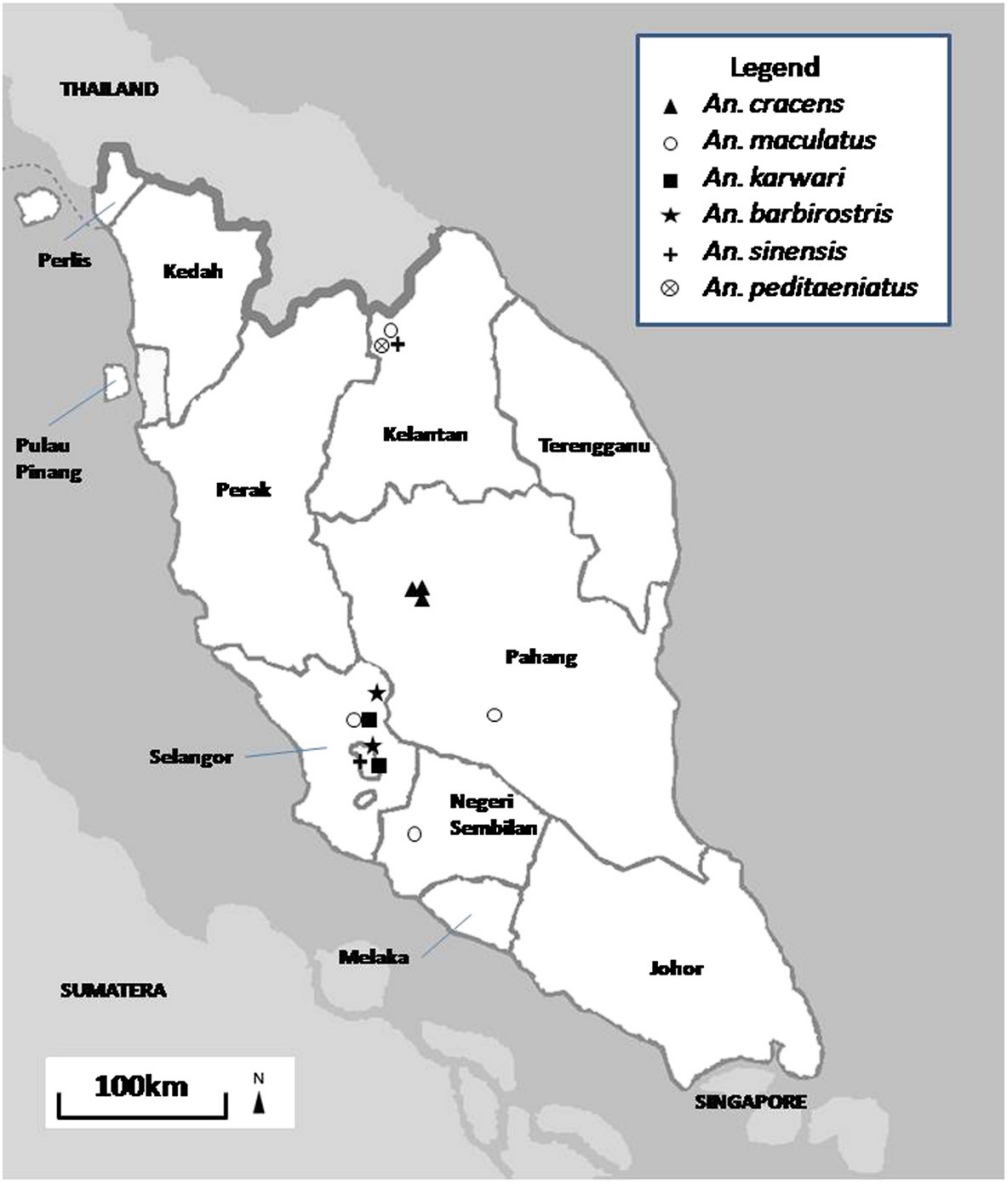
**Map showing locations of fieldwork across Peninsular Malaysia, with *Anopheles *** **spp. catchment being shown with different legends.**

In total, 133 *Anopheles* mosquitoes consisting of six different species were collected from eight different locations across Peninsular Malaysia (Figure 1, Additional file 1: Table S1). These *Anopheles* mosquitoes were collected from suburban, rural and forested areas. The collected *Anopheles* species were *An. cracens*, *An. maculatus*, *An. karwari*, *An. barbirostris*, *An. sinensis* and *An. peditaeniatus*. All specimens were negative for *Plasmodium* sporozoite infection from dissection and PCR.

**Figure 2:**
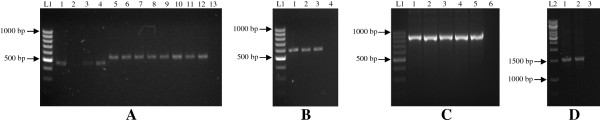
**Gel pictures showing PCR amplicons generated from the six *Anopheles *species using ITS2A and ITS2B primers.** Different species show amplicons of different sizes. Lane L1 denotes lane with DNA ladders of 100 bp whereas lane L2 indicates lane with DNA ladders of 1000 bp. The right-most lane of each picture represents the “no-template control”. Agarose gels of 1.5 % **(A-C)** and 1.3 % **(D)** were used. The amplicons of *An. maculatus* (lane 1 – 4) and *An. karwari* (lane 5 – 12) were shown to be 460 bp and 526 bp respectively **(A)**. For *An. peditaeniatus* (lane 1) and *An. sinensis* (lane 2 & 3), amplicons were of 583 bp and 575 bp respectively **(B)**. Specimens of *An. cracens* (lane 1 – 5) were shown to yield amplicons of 855 bp **(C)**. Amplicons of *An. barbirostris* specimens (lane 1 & 2) were 1576 bp long **(D)**.

**Figure 3:**
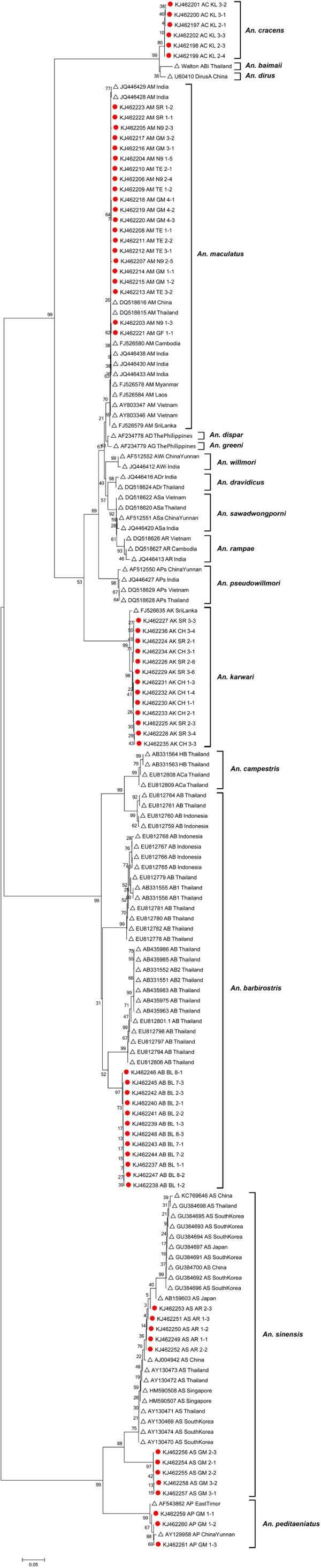
**Phylogenetic tree based on ITS2 rDNA gene sequences of *Anopheles *spp. By using the Neighbor-Joining (bootstrap = 1000).** Sequences marked with orange dots are sequences yielded from this study, whereas the rest are sequences from GenBank. GenBank accession number is given after each isolate’s name.

From these mosquitoes, 65 ITS2 rDNA sequences were obtained. Amplicons with consistently distinct sizes were yielded from each species, with *An. cracens* being 855 bp; *An. maculatus* being 460 bp; *An. karwari* being 526 bp; *An. barbirostris* with 1576 bp; *An. sinensis* yielding 575 bp; and *An. peditaeniatus* being 583 bp (Figure 2). Sequence alignment of *An. barbirostris* was difficult due to several reasons. Firstly, there are many variable length nucleotide sequence repeats within ITS2 rDNA of *An. barbirostris*. Besides, the *An. barbirostris* ITS2 rDNA sequence range covered by ITS2A and ITS2B primer set is too long (1576 bp), causing low resolution of nucleotide reading in both one-way and two-way sequencing methods. As a result, another forward sequencing primer, ITS2M (5’ GCG TGG TCT ACT AGT TAG AC 3’) was designed to target nucleotide sequences in the middle range of the amplicon, thus increasing the nucleotide reading resolution in sequencing. For phylogenetic comparison between anophelines across Asian regions, anopheline ITS2 sequences from other countries that are available in GenBank were used for phylogeny tree construct and analysis (Figure 3).

**Figure 4:**
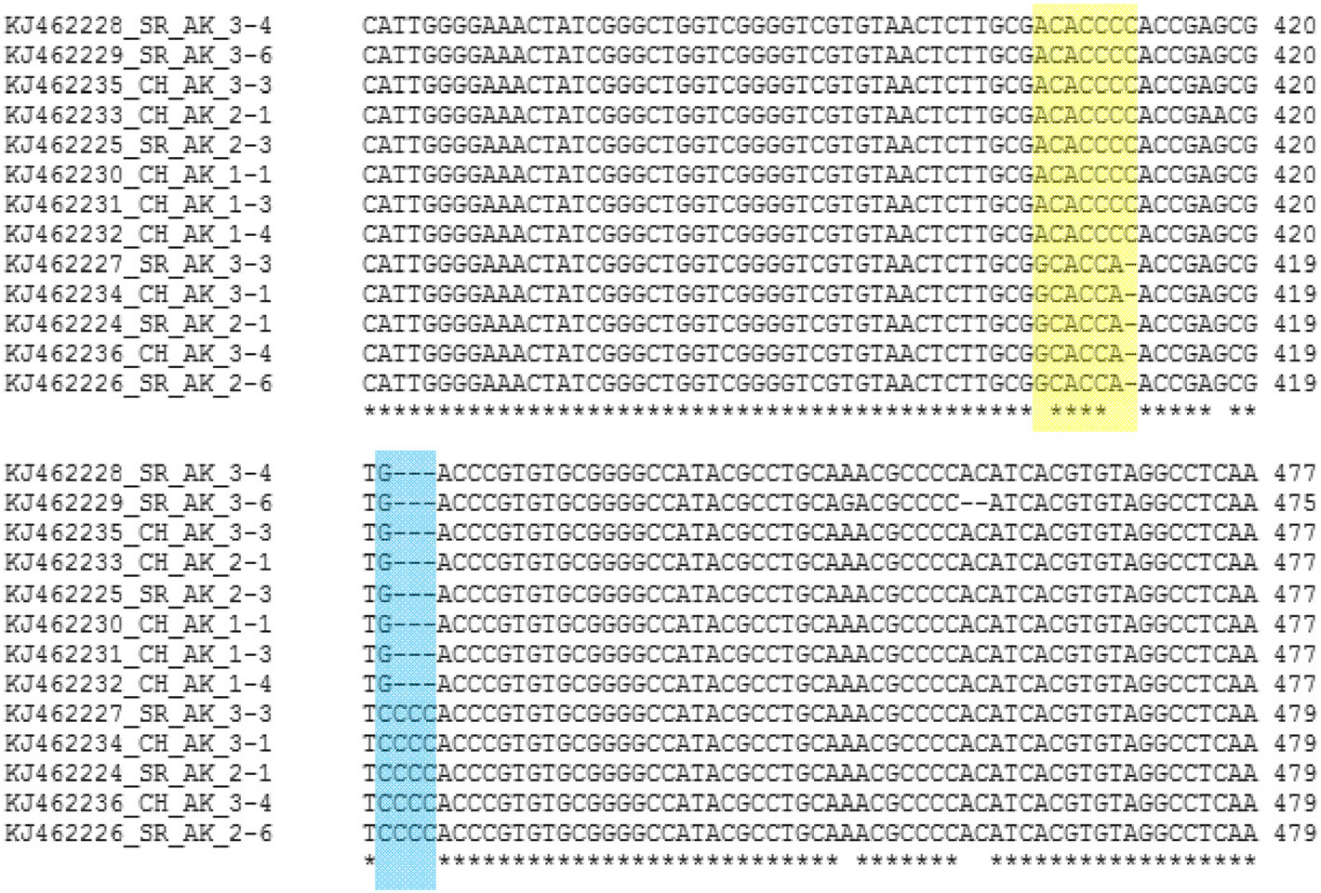
**Sequence alignment of *An*. *karwari *from this study using ClustalW software.** The shaded areas (indicated by yellow and blue) show the region with distinct sets of nucleotide sequence.

By analyzing sequences obtained from cloning, coupled with reference sequences from GenBank, point mutations were found in all collected species, with *An. karwari* showing the highest prevalence (22 point mutations detected), followed by *An. barbirostris* (19 point mutations detected), *An. maculatus* (12 point mutations detected), *An. cracens* (10 point mutations detected), *An. peditaeniatus* (6 point mutations detected), *An. sinensis* from northern Peninsular Malaysia (3 point mutations detected), and *An. sinensis* from central Peninsular Malaysia (1 point mutation detected). In addition, other forms of mutations such as deletion (in all six species studied), insertion (in *An. cracens*, *An. barbirostris*, *An. karwari* and *An. sinensis*), duplication (in *An. cracens* and *An. barbirostris*) and small tandem repeats (in all six species studied) were found as well. Interestingly, two sets of ITS2 rDNA nucleotide sequences (with size difference of 2 bp) with distinctive patterns of mutations (insertions and deletions) were found in each specimen of *An. karwari* (Figure 4).

Based on sequence alignment and comparison, ITS2 rDNA sequences from the collected *An. cracens* showed no obvious nucleotide sequence variation. Due to lack of ITS2 rDNA sequences of *An. cracens* from other countries, phylogenetic analysis on this particular species could not be conducted. Nevertheless, ITS2 rDNA sequences of two related species [12,49], *An. dirus* (formerly known as *An. dirus* A) and *An. baimaii* (formerly known as *An. dirus* D) from GenBank were recruited for the phylogenetic analysis. As expected, *An. cracens* is closely related but distinct from *An. dirus* and *An. baimaii*. Likewise, obvious geographical clustering was not detected among *An. maculatus* from Peninsular Malaysia, as well as with those of other Asian countries. Nevertheless, *An. maculatus* ITS2 rDNA sequences show clear difference but close relationship with other members from *An. maculatus* complex (*An. dispar*, *An. greeni*, *An. sawadwongporni*, *An. rampae*, *An. dravidicus*, *An. pseudowillmori* and *An. willmori*) [31,50-52]. From the phylogenetic tree, *An. karwari* shows a relatively close relationship with *An. maculatus* group. This should not be too surprising since *An. karwari* shows high resemblance to *An. maculatus* in morphological features. For *An. barbirostris*, all five clades of *An. barbirostris* complex were included for the analysis. The *An. barbirostris* specimens collected from this study were most closely related to Clade IV. By comparison, members from Clade I, as well as Clade V (also known as *An. campestris*) are the most distantly apart from *An. barbirostris* specimens collected from this study.

As mentioned earlier, the collected *An. karwari* showed distinct patterns of mutations. Indeed, the mutation pattern seen in the collected Peninsular Malaysian *An. karwari* was different from that of Sri Lankan *An. karwari* (sequences from GenBank) [53]. Meanwhile, *An. barbirostris* collected were related, but distinct from the Thai and Indonesian *An. barbirostris*. Interestingly, *An. sinensis* obtained from two distantly apart locations in Malaysia (northern Peninsular Malaysia and central Peninsular Malaysia) were depicted as two distinctive clusters, with the cluster originated from central Peninsular Malaysia situated closer to clusters from the oriental region (South Korea, China and Japan) and other Southeast Asian countries (Thailand and Singapore). Only one *An. peditaeniatus* was collected throughout the study. Since the *An. peditaeniatus* ITS2 rDNA sequences from other countries that are archived in GenBank are too short [54], phylogenetic comparison of the *An. peditaeniatus* population could not be conducted.

## Discussion

In this study, *Plasmodium* sporozoite-positive *Anopheles* were not found. This may be due to several reasons. Firstly, the population of *Anopheles* mosquitoes infected with *Plasmodium* sporozoites may be very small, and such a small portion of infected *Anopheles* is likely to be missed in the fieldwork. Indeed, the malaria transmission in Peninsular Malaysia has been reduced to very low levels and malaria cases only occur sporadically [55]. Hence the probability of finding *Anopheles* positive with *Plasmodium* sporozoite is also low. Coupled with the relatively small sampling size of this study, the chance of getting an infected *Anopheles* mosquito is even smaller. In addition, the exact geographical source of infection reported by patients to the District Health Offices may not be accurate.

The sizes of amplicons obtained from this study vary from one species to another, indicating a high rate of insertion and deletion (INDEL) mutations on this gene. Four species (*An. maculatus*, *An. karwari*, *An. sinensis* and *An. peditaeniatus*) fall into the range of 460 to 583 bp, whereas *An. cracens* and *An. barbirostris* yield much larger amplicons (855 and 1576 bp respectively). As mentioned earlier, *An. barbirostris* has many variable length nucleotide sequence repeats within its ITS2 rDNA. This finding is parallel to those reported previously [15,56]. For *An. cracens*, the larger size amplicon is due to duplication of nucleotide sequences. Indeed, the yield of larger amplicons with ITS2 primers is also found in other members of *An. leucosphyrus* group (e.g. *An. dirus* and *An. baimaii*) [49,57].

Point mutations were detected in all species of anophelines collected in this study, albeit with different rates. It is important to note that “point mutation-like” single nucleotide differences may arise from Taq polymerase and sequencing errors. Nevertheless, such probability was ruled out in this study. Prior to this study, an independent experiment was conducted on a well conserved gene using the same Taq polymerase used in this study (data not shown). The conserved gene cloned in plasmid was amplified using the same Taq polymerase and cloned into pGEM®-T vector. Three clones were selected for sequencing. Each set showed 100% identity with the original recombinant plasmid carrying the conserved gene sequence. This indicates that the Taq polymerase used in this study is reliable for nucleotide sequence analysis.

The lack of apparent nucleotide sequence variation among ITS2 rDNA sequences of the collected *An. cracens* may be due to the fact that all *An. cracens* specimens were collected from separate, but adjoining forested areas within the state of Pahang. Therefore, the genetic interchange between the *An. cracens* from these locations is not impeded. On the other hand, the presence of two distinct sets of ITS2 rDNA sequences in each *An. karwari* is possibly due to the multiple copies of ITS2 within rDNA of each mosquito, where two distinct mutations arise. In this study, sequences archived by previous study on Sri Lankan *An. karwari* were recruited for phylogenetic analysis [53]. The analyses showed that the mutation patterns of Peninsular Malaysian *An. karwari* were different from that of the Sri Lankan *An. karwari*. Interestingly, the Sri Lankan *An. karwari* was shown to be distinct from *An. karwari* from other Asian countries (India, Myanmar and Cambodia) based on mitochondrial DNA analysis [53]. Therefore, it is possible that the mutation pattern found in *An. karwari* collected from this study would bear more resemblance to that of *An. karwari* from those countries, as compared to the Sri Lankan *An. karwari*. However, further studies are needed to verify this observation.

One critical difficulty in phylogenetic tree construct for this study is the inadequate amount of usable nucleotide sequences from other countries. Some of the archived sequences are too short while some other sequences are of different gene regions that offer small, if not zero overlapping with our nucleotide sequences. Indeed, such difficulty is also mentioned by a previous study [29]. Nevertheless, a phylogenetic tree was constructed by using the available resources. Based on the phylogenetic tree constructed, all *Anopheles* mosquitoes from Peninsular Malaysia show close phylogenetic relationship with the same species of *Anopheles* from Asian regions, especially the mainland countries. The relatively closer relationship with nearer mainland countries than the Asian islets shows that geographical isolation confers distinctive genetic pools to the mosquitoes on the islets, resulting in bigger genetic difference with those from the mainland. For instance, *An. greeni* and *An. dispar* are two species on the Philippines that are evolved from *An. maculatus* due to long period of geographical isolation [50]. Nevertheless, islets that are very close to Peninsular Malaysia like Singapore still have mosquitoes that are genetically close to Peninsular Malaysian mosquitoes [15,58]. At the same time, places from the mainland that are well isolated by mountains harbour mosquitoes with distinctive genotypes as well. This is well exemplified by the phylogenetic analysis of *An. barbirostris* in this study. The *An. barbirostris* captured from this study showed close relationship with those from Muang District of Trat Province, Thailand [15], which is located at the Gulf of Thailand. Meanwhile, these Peninsular Malaysian mosquitoes are quite different from the *An. barbirostris* of Indonesian Sumatra, as well as those from Sa Kaeo Province and Mae Hong Son Province of Thailand [15]. As Indonesian Sumatra is separated from Peninsular Malaysia by the Strait of Malacca while Sa Kaeo and Mae Hong Son are valleys isolated by mountains and thick forests, it is not surprising that the *Anopheles* mosquitoes from these locations are more distantly apart from the Peninsular Malaysian Anopheline mosquitoes in phylogenetic analysis.

Interestingly, *An. sinensis* collected from Kelantan (northern Peninsular Malaysia) were distinct from *An. sinensis* of central Peninsular Malaysia and other Asian countries. Such difference suggests development of distinct geographic races or allopatric speciation evolvement of these *An. sinensis* populations due to long periods of complete geographical isolation. The two sampling locations are separated by highlands such as the Tahan Range and Titiwangsa Mountains (Sankalakhiri Range). Geographical segregation (vicariance) prevents genetic flow, interchange and interaction between population pools of a same species. Consequently, these distinguished pools of mosquito populations evolve and show genetic differences. Indeed, *An. belenrae* and *An. kleini* were upgraded to distinct species from the South Korean *An. sinensis* population via ITS2 rDNA study [24,59]. Nevertheless, the status of this group of Kelantanese *An. sinensis* remains to be validated. More gene markers such as ND6, COI and COII should be tested to verify its actual taxonomical status [15,17].

Another interesting point worth mentioning here is the capture of *An. barbirostris* via BLC technique from this study. In Peninsular Malaysia, *An. barbirostris* is regarded as a zoophilic mosquito [60]. However, this species was collected easily using BLC in locations adjacent to housing areas. This suggests that the feeding behaviour of *An. barbirostris* has changed and adapted to human blood feeding. Since this species is one of the major malaria vectors in Timor Leste [61], more attention should be given to study the sporozoite infectivity of *An. barbirostris* in Peninsular Malaysia.

## Conclusion

Based on this study, *Anopheles* mosquitoes from Peninsular Malaysia show close evolutionary relationship with the Asian anophelines. Nevertheless, genetic differences can be seen between the Peninsular Malaysian *Anopheles* and the *Anopheles* of certain places of this region due to geographical segregation. In addition, populations of some *Anopheles* mosquitoes in Peninsular Malaysia show divergent evolutionary progress, exemplified by the emergence of distinct cluster of *An. sinensis* populations due to geographical segregation, suggestive of allopatric speciation.

## Competing interests

The authors declare that they have no competing interests.

## Authors’ contributions

JSS, AA, JJ, NMAA and KAB conducted fieldwork for mosquito collection. JSS conducted and processed molecular diagnoses. JSS, WCL and MYF collected, analyzed and interpreted the data. JSS, WCL and MYF constructed and analyzed phylogenetic tree. WCL, JSS, MYF and YLL arranged the data, conceptualized and prepared the manuscript. All authors read and approved the final manuscript.

## Supplementary Material

Additional file 1: Table S1 Coordinates of locations with *Anopheles* catchment. Click here for file
