# Supplementary material for: Phylogenetic study of six species of Anopheles mosquitoes in Peninsular Malaysia based on inter-transcribed spacer region 2 (ITS2) of ribosomal DNA
Source: Parasit Vectors. 2014 Jul 3;7:309. doi: 10.1186/1756-3305-7-309 (PMC4094596; doi:10.1186/1756-3305-7-309)
Supplement: Additional file 1: Table S1 — Coordinates of locations with Anopheles catchment. [file 1756-3305-7-309-S1.docx]

| ***Anopheles* species** | **Coordinate**  **(Latitude)** | **Coordinate (Longitude)** | **Numbers collected** |
| --- | --- | --- | --- |
| *An. cracens* | 4° 12' 39.4848“N | 101° 52' 27.9582“E | 6 |
|  | 4° 12' 34.9776“N | 101° 52' 30.6264“E | 1 |
|  | 4° 12' 34.4874“N | 101° 52' 26.7564“E | 3 |
| *An. maculatus* | 2° 56' 22.6038“N | 102° 4' 32.3288“E | 2 |
|  | 3° 37' 0.0012“N | 102° 20' 59.9994“E | 3 |
|  | 5° 45' 16.8042“N | 101° 44' 48.1914“E | 13 |
|  | 3° 22' 38.2182“N | 101° 38' 14.661“E | 1 |
| *An. karwari* | 3° 6' 19.803“N | 101° 43' 45.5736“E | 9 |
|  | 3° 22' 38.2182“N | 101° 38' 14.661“E | 39 |
| *An. barbirostris* | 3° 34' 43.3158“N | 101° 44' 4.9452“E | 19 |
|  | 3° 15' 45.7338“N | 101° 37' 35.3676“E | 12 |
| *An. sinensis* | 5° 45' 16.8042“N | 101° 44' 48.1914“E | 2 |
|  | 3° 7' 5.9298“N | 101° 34' 48.3348“E | 2 |
| *An. peditaeniatus* | 5° 45' 16.8042“N | 101° 44' 48.1914“E | 1 |
